# Pediatric delirium is associated with increased brain injury marker levels in cardiac surgery patients

**DOI:** 10.1038/s41598-022-22702-2

**Published:** 2022-11-04

**Authors:** Yujiro Matsuishi, Haruhiko Hoshino, Yuki Enomoto, Nobutake Shimojo, Muneaki Matsubara, Hideyuki Kato, Bryan J. Mathis, Kojiro Morita, Yuji Hiramatsu, Yoshiaki Inoue

**Affiliations:** 1 Neuroscience Nursing, St. Luke’s International University, Tokyo, Japan; 2 Artificial Intelligence Laboratory, University of Tsukuba, Tsukuba, Ibaraki Japan; 3 Adult Health Nursing, Department of Nursing, International University of Health and Welfare, Narita, Japan; 4 Department of Emergency and Critical Care Medicine, Faculty of Medicine, University of Tsukuba, Tsukuba, Ibaraki Japan; 5 Department of Pediatrics, University of Tsukuba Hospital, Tsukuba, Ibaraki Japan; 6 Department of Cardiovascular Surgery, University of Tsukuba, Tsukuba, Ibaraki Japan; 7 International Medical Center, University of Tsukuba Hospital, Tsukuba, Ibaraki Japan; 8 Global Nursing Research Center, Graduate School of Medicine, The University of Tokyo, Tokyo, Japan

**Keywords:** Neuroscience, Health care, Medical research, Paediatric research

## Abstract

Despite global consensus on the importance of screening pediatric delirium, correlations between pediatric delirium during acute brain injury and adult delirium are unclear. Therefore, we hypothesized that similar pediatric biomarkers reflect acute brain injury as in adult delirium. We observed pediatric cardiac surgery patients from neonatal age to 18 years, who were admitted to our pediatric intensive care unit after cardiovascular operations between October 2019 to June 2020, up to post-operative day 3 (4 days total). We recorded age, sex, risk score (Risk Adjustment in Congenital Heart Surgery [RACHS-1]), midazolam/dexmedetomidine/fentanyl dosage, and pediatric Sequential Organ Failure Assessment (pSOFA). Richmond Agitation-Sedation Scale (RASS), Cornell Assessment of Pediatric Delirium (CAPD), Face, Leg, Activity, Consolability (FLACC) behavioral scale, and Withdrawal Assessment Tool (WAT-1) scales were used and serum sampling for neuron specific enolase (NSE) was conducted. Consciousness status was considered hierarchical (coma > delirium > normal) and associations between conscious status and NSE were conducted by hierarchical Bayesian modeling. We analyzed 134 data points from 40 patients (median age 12 months). In the multi-regression model, NSE was positively associated with coma [posterior odds ratio (OR) = 1.1, 95% credible interval (CrI) 1.01–1.19] while pSOFA [posterior OR = 1.63, 95% CrI 1.17–2.5], midazolam [posterior OR = 1.02, 95% CrI 1.01–1.04], and dexmedetomidine [posterior OR = 9.52, 95% CrI 1.02–108.85] were also associated. We also evaluated consciousness state probability at each NSE concentration and confirmed both that consciousness was hierarchically sorted and CAPD scores were also associated with NSE [posterior OR = 1.32, 95% CrI 1.09–1.58]. “Eye contact” (r = 0.55) was the most correlated component with NSE within the pain, withdrawal syndrome, and PD items. PD within the hierarchy of consciousness (coma, delirium, normal) and CAPD scores are associated with brain injury marker levels. Using pediatric delirium assessment tools for monitoring brain injury, especially eye contact, is a reliable method for observing PD.

## Introduction

Delirium is the most commonly treated neuropsychological complication after cardiac operation in adult patients, with a reported prevalence of between 26 to 52%^1^, and is independently associated with negative outcomes such as increased length of stay^2^, mortality^3^, and cognitive impairment^2^. Recently, delirium has been recognized as a form of brain dysfunction and its association with biomarkers, such as s100β, have been reported^4^. With this revelation, the concept of measuring freedom from both delirium and coma was developed to fully encompass the spectrum of cardiac surgery-related neurological complications ^5,6^.

In pediatric settings, pediatric delirium (PD) has been judged as a serious issue and diverse assessment tools, such as the Pediatric Confusion Assessment Method for the ICU (pCAM-ICU)^7^, Preschool CAM-ICU (psCAM-ICU)^8^, Sophia Observation withdrawal Symptoms-Pediatric Delirium scale (SOS-PD)^9^, and Cornell Assessment of Pediatric Delirium (CAPD)^10^, were established to quantify delirium-associated factors. However, evaluating PD is more difficult than delirium in adults even if diagnosed by the same basic criteria of the 5th Diagnostic and Statistical Manual of Mental Disorders (DSM-5)^11^. This creates an issue because pediatric patients do not yet have a full range of cognition and so psychiatric assessments become more subjective than objective, reducing validity and specificity. Moreover, as evaluating PD is behaviorally based, pain and iatrogenic withdrawal syndrome (IWS) may confound PD diagnoses and discrimination between pain, IWS, and PD requires valid external indicators.

With regard to PD and pain, it is essential to clearly distinguish between these states in order to optimize therapeutic interventions in postoperative care for clinicians. A previous study already revealed differences in the timing of emergence between PD and pain but the results were based on screening tools that are currently unexplored with respect to molecular mechanisms^12^. Between PD and IWS, In the STROKE study, authors reported that 47% of participating patients had IWS^13^ but concerns of overestimation came from the lack of delirium assessment in that study^14^. Symptoms of both diseases overlap because IWS thought to be a cause of delirium^13^, an idea that remains controversial.

Therefore, since delirium has been classified as brain dysfunction in adults, it may be possible to also evaluate similar associations between brain injury biomarkers and delirium in pediatric patients. Several studies revealed the increase of glial fibrillary acidic protein (GFAP) after cardiopulmonary bypass in the pediatric population^15,16^, indicative of brain injury after CPB. Additionally, several brain injury biomarkers reported in pediatric patients include serum calcium-binding protein β (S-100β) and neuron-specific enolase (NSE). A previous meta-analysis revealed NSE had a moderate predictive value for brain injury in pediatric patients^17^ and that NSE has been reported as reflective of brain injury after cardiac surgery in pediatric patients^18^. Moreover, serum NSE levels putatively function as a predictor of outcome in comatose cardiac-arrest survivors^19^ while a systematic review and meta-analysis showed mortality and unfavorable outcomes were significantly associated with greater NSE concentrations^20^. However, time course changes of these two biomarkers, especially in pediatric patients, are still not fully revealed. Notably, a previous study revealed time course changes in S-100β and NSE after hypoxia in a newborn pig model, revealing a significant difference in NSE between the control/mild insult group and severe insult group (but not S-100β) after 24 h^21^. From this aspect, NSE can be considered a reactive biomarker to reflect damage compared with slower-reacting S-100β after brain injury in pediatric patients.

We thus establish the hypothesis that pediatric patients who undergo CPB and have PD will have changes in acute brain injury biomarkers reflective of status.

## Material and methods

### Patient selection

This study is an observational prospective study that included patients from neonatal age to 18 years who underwent cardiovascular operations between October 2019 to June 2020 at the University of Tsukuba Affiliated Hospital. Patients were excluded if they were diagnosed with brain abnormalities, stroke, and/or epilepsy before surgery. Cases featuring post-surgical cardiac arrest and/or use of extracorporeal membrane oxygenation (ECMO) during the study period were excluded to reduce outcome bias. We also excluded patients who received muscle relaxants continuously on observation days because of difficulties in estimating consciousness. In this study, we evaluated patients up to post-operative day (POD) 3 (4 days total) and terminated observation when the patients were decannulated from an arterial line catheter or discharged from the pediatric intensive care unit (PICU). In our practice, patients are decannulated when their hemodynamics are stable and not in acute phase. We therefore stopped blood sampling at that time as extra injections and blood sampling for these pediatric patients is ethically unreasonable. The Institutional Review Board (IRB) of the University of Tsukuba Affiliated Hospital approved the present study (#1-014) and written, informed consent was obtained from the parents or legal guardians of all patients prior to surgery.

### Preoperative period

All patients were admitted before the operation and all operations were planned. No patients were previously hospitalized for other operations and were living at home before their procedures.

### Data collection

We recorded baseline demographic data, including age, sex, and risk score as measured by the Risk Adjustment in Congenital Heart Surgery (RACHS-1)^22^ tool which classifies surgical procedures into six categories based on mortality risk. RACHS-1 was previously validated by large multi-institutional data sets^23–25^. We also recorded midazolam and/or dexmedetomidine dosage plus the severity of organ dysfunction as calculated by the pediatric Sequential Organ Failure Assessment (pSOFA) as post-operative daily data. The pSOFA for pediatric critically ill patients was created using age-adjusted criteria and validated with an original study that included not only assessments of septic patients but also cardiovascularly compromised and post-operative recovery patients^26^. A previous study revealed the application of former SOFA scores after cardiac surgery^27^. In our practice, we use only benzodiazepine and dexmedetomidine for sedation and pSOFA is an assessment score for pediatric patients that was validated retrospectively in critically ill pediatric patients^26^.

### Sedation and delirium assessment

Sedation depth and delirium were assessed using the Richmond Agitation—Sedation Scale (RASS)^28^ and the Cornell Assessment of Pediatric Delirium (CAPD)^10^ for the up-to-3-day post-operative (up to 4 days total) study period. RASS is a widely used sedation scale originally developed for adults but was recently validated in pediatric patients^29^. The CAPD scale is a standard assessment scale for PD appropriate for a wide age range (neonate to 21 years old) and we recently validated the Japanese version in pediatric patients^30^.

### Related symptoms: pain and withdrawal symptom assessment

We also assessed related symptoms (pain and withdrawal symptoms) by the Face, Leg, Activity, Consolability (FLACC) behavioral scale^31,32^ and the Withdrawal assessment tool (WAT-1). The FLACC behavioral scale is a standard tool for evaluating pediatric pain and was validated in PICU settings. WAT-1 is also a standard tool for evaluating pediatric IWS and was validated in PICU settings. In PICU settings, oft- used benzodiazepine and opioids are risk factors for IWS^33^, thus we evaluated IWS symptoms after weaning of benzodiazepine and/or opioids for 72 h by using WAT-1^34^. Both tools are recommended by the European Society of Paediatric and Neonatal Intensive Care for clinical evaluation of pain, sedation, withdrawal and delirium assessment in critically ill pediatric patients^35^.

### Postoperative period evaluation of pain, sedation, withdrawal and delirium assessment

We established a Pediatric patients Outcome ReCOrding system (PORCO) and all evaluations were analyzed by researchers once a day during routine daytime meetings. The researchers previously established Japanese versions of the FLACC^32^, CAPD^30^, and WAT-1 instruments^36^.

### Brain injury biomarker

We assayed the brain‑specific isoform of enolase known as neuron-specific enolase (NSE) that has been reported as reflective of brain injury after cardiac surgery in pediatric patients^18^. Serum NSE levels as predictor of outcome in comatose cardiac-arrest survivors^19^, and systematic review and meta-analysis shows mortality and unfavorable outcome were significantly associated with greater NSE concentrations^20^. Several previous studies also reported the association between elevated NSE and post-operative delirium in cardiac surgery in both pediatric patients and adults^37,38^.

Therefore, serum NSE level thought to be the reliable marker for brain injury. We collected blood samples after return to the PICU at POD:0 and around 6 a.m. from POD:1 up to POD:3 from an arterial line catheter. All blood samples were centrifuged at 1000×*g* for 15 min at room temperature and stored at − 60 °C until analysis by serum sampling. Commercially available ELISA kits were used to measure serum concentrations of human NSE (BioVendor Laboratory Medicine, Inc., Czech Republic).

### The hierarchical state of consciousness model

Recent studies have positioned states of consciousness as coma, delirium, and non-coma/non delirium (hereafter normal). Therefore, the concept of non-delirium, coma-free days^39–41^ and statistical methods to estimate the odds of risk factors in changing these three states of consciousness (daily transition model) have been reported in existing studies^42^. Following these lines of research, the present study also assumed that states of consciousness exist in the order of coma, delirium, and normal. Coma is defined as deep sedation (score: − 4) or coma (score: − 5) using the RASS sedation scale while delirium is defined as a patient who is not comatose and is assessed positive for delirium with the CAPD. Normal is defined as a patient who is neither comatose nor delirium positive. The recommended method for defining coma and delirium is in the CAPD assessment.

### Statistical analysis

#### Multi-faceted statistical modeling

We approached the various aspects of the study question by establishing four models as follows:

- Model 1: Multivariate modeling to evaluate the association between NSE and hierarchical consciousness status. This model tests if NSE is associated with hierarchical consciousness status.
- Model 2: Multivariate modeling to confirm a hierarchical association within consciousness status. This model examines if consciousness hierarchically associates with NSE.
- Model 3: Multivariate modeling to evaluate the association between NSE and CAPD scores without coma data. This model tests if CAPD scores are associated with NSE.
- Model 4: Correlation analysis to evaluate the association between NSE and pain, delirium, and withdrawal symptoms.

#### Sample size calculations

We applied Bayesian modeling, which requires a large sample size for the estimation, and calculated the correct sample size for reference values using G * Power 3.1 software^43^. Assuming a linear regression and an effect size of f^2^ = 0.15 (moderate effect), we determined that a sample size of 124 observations would be required for a significance level (α) of 0.01 and test power (1 − β) of 0.95, reflecting a very rigorous estimation.

#### Model structure for multivariate modeling

The outcome of interest in Model 1 was the state of hierarchical consciousness status (coma, delirium and normal as ordered) and the dependent factor was serum concentration of NSE. To adjust our model, the following additional covariates were chosen a priori: sex, age, RACHS-1, dose of benzodiazepine, dose of dexmedetomidine and pSOFA (without the central nervous system component). To adjust patient demographic characteristics, we chose sex and age as covariates. RACHS-1 was used to adjust operation severity and pSOFA (without the central nervous system component) was used to adjust post-surgical daily severity. All sedative agents (benzodiazepine and dexmedetomidine) and opioids (only fentanyl was used for our cohort) were also used to adjust sedation status. These covariates were also applied to Models 2 and 3 but not Model 4 because of the correlation analysis.

### Statistical estimation

As we previously reported^44^, we applied Bayesian modeling^45,46^ for this study.

Hierarchical Bayesian modeling was applied using the No-U-turn sampler (NUTS), an extension of the Hamiltonian Monte Carlo (HMC) algorithm for Markov chain Monte Carlo. All iterations were set to 10,000, the burn-in sample to 5,000, and the number of chains to 5. We checked the modeling assumption by setting values for effective sample size as Rhat and the Monte Carlo Standard Error (MCSE)/standard deviation (sd). An MCSE/sd less than 10% and a Rhat for all parameters less than 1.1^47^ indicated a good estimation for the model. We report the 95th percentile interval as a 95% credible interval (Crl). All statistical analyses were performed with R. (The R Foundation for Statistical Computing, Vienna, Austria).

### Ethics approval

This study was carried out under laws equivalent to or derived from the principles of the Declaration of Helsinki of 1975, and the Institutional Review Board (IRB) of the University of Tsukuba Affiliated Hospital approved the present study (#R01-014: Pediatric postoperative cardiac surgery delirium in relation to oxygenation and neuro-sleep system substances, 2019/6/25 approved.) and written, informed consent was obtained from the parents or legal guardians of all patients prior to surgery. All the procedures were followed in accordance with the ethical standards of the responsible committee.

## Results

### Patient characteristics

We enrolled 53 patients but excluded 13 patients for the following reasons: nine who received muscle relaxants during the study period, one who received ECMO for cardiac pulmonary arrest, and three who were in violation of our blood sampling protocol. Thus, we analyzed a total of 134 data points from 40 patients for this study. We show the demographic characteristics of participating patients in Table 1. The median age of the participants was 12 (range: 6 to 61) months, with ages ranging from 2 months to 15 years. Of 40 patients total, 13% were 0–3 months old, 40% were 3–12 months old, 22% were 1–5 years old, and 25% were 6–18 years old. The proportion of male and female patients was equal (50%). All operations were considered class 4 as estimated by RACHS-1 scoring. In category 1 all procedures were atrial septal defect (ASD) closings (8 cases) while, in category 2, most of the cases (15 cases) were ventricular septal defect (VSD) closings. For category 3, most cases (6 cases) were total cavopulmonary connections (TCPC), including extracardiac TCPC) After surgery, 60% of patients received midazolam at POD:0 and this number decreased to 36% by POD:3. Likewise, 90% of patients received post-surgical dexmedetomidine at POD:0, decreasing to 52% by POD:4. Similarly to the sedative agents, 95% of patients received post-surgical fentanyl at POD:0, decreasing to 60% by POD:4. The severity of organ scores without the consciousness component, as estimated by pSOFA, gradually decreased by POD:4 (POD:0 median of 4, POD:4 median of 3).

**Table 1:** Baseline characteristics of the patients.

Variable	Participating patients N = 40
Age (months) (IQR)	12 (6, 61)
**Age categories, n (%)**	
0–3 months	5 (13)
3–12 months	16 (40)
1–5 years	9 (22)
6–18 years	10 (25)
Female, n (%)	20 (50)
**RACHS-1, n (%)**	
Category 1	8 (20%)
Category 2	23(58%)
Category 3	9 (22%)
pSOFA, score, ± SD	4 (2,6)
**Average**	
**Total days**	
POD 0	4 (3, 6)
POD 1	3 (2, 5)
POD 2	3 (2, 6)
POD 3	3 (1, 6)
**Midazolam**	
**Average dose by day**	
Total days, μg/kg/day ± SD	40.5 ± 61.7
POD 0, μg/kg/day ± SD	43.6 ± 55.2
POD 1, μg/kg/day ± SD	31.8 ± 54.8
POD 2, μg/kg/day ± SD	46.5 ± 74.5
POD 3, μg/kg/day ± SD	42.7 ± 68.2
**Proportion of receiving patients**	
POD 0, n/total patients each day, (%)	24/40 (60)
POD 1, n/total patients each day, (%)	14/40 (35)
POD 2, n/total patients each day, (%)	11/29 (37)
POD 3, n/total patients each day, (%)	9/25 (36)
**Dexmedetomidine**	
**Average dose by day**	
Total days, μg/kg/day ± SD	0.39 ± 0.32
POD 0, μg/kg/day ± SD	0.50 ± 0.29
POD 1, μg/kg/day ± SD	0.39 ± 0.27
POD 2, μg/kg/day ± SD	0.33 ± 0.31
POD 3, μg/kg/day ± SD	0.13 ± 0.32
**Proportion of receiving patients**	
POD 0, n/total patients each day, (%)	38/40(95)
POD 1, n/total patients each day, (%)	36/40 (90)
POD 2, n/total patients each day, (%)	22/29 (76)
POD 3, n/total patients each day, (%)	13/25 (52)
**Fentanyl**	
**Average dose by day**	
Total days, μg/kg/day ± SD	0.48 ± 0.48
POD 0, μg/kg/day ± SD	0.76 ± 0.49
POD 1, μg/kg/day ± SD	0.51 ± 0.48
POD 2, μg/kg/day ± SD	0.34 ± 0.38
POD 3, μg/kg/day ± SD	0.14 ± 0.25
**Proportion of receiving patients**	
POD 0, n/total patients each day, (%)	38/40(95)
POD 1, n/total patients each day, (%)	36/40 (90)
POD 2, n/total patients each day, (%)	20/29 (68)
POD 3, n/total patients each day, (%)	15/25 (60)

pSOFA: pediatric-sequential organ failure assessment score ,POD: Post-operative day, RACHS-1: Risk-Adjusted Congenital Heart Surgery-1.

**Figure 1:**
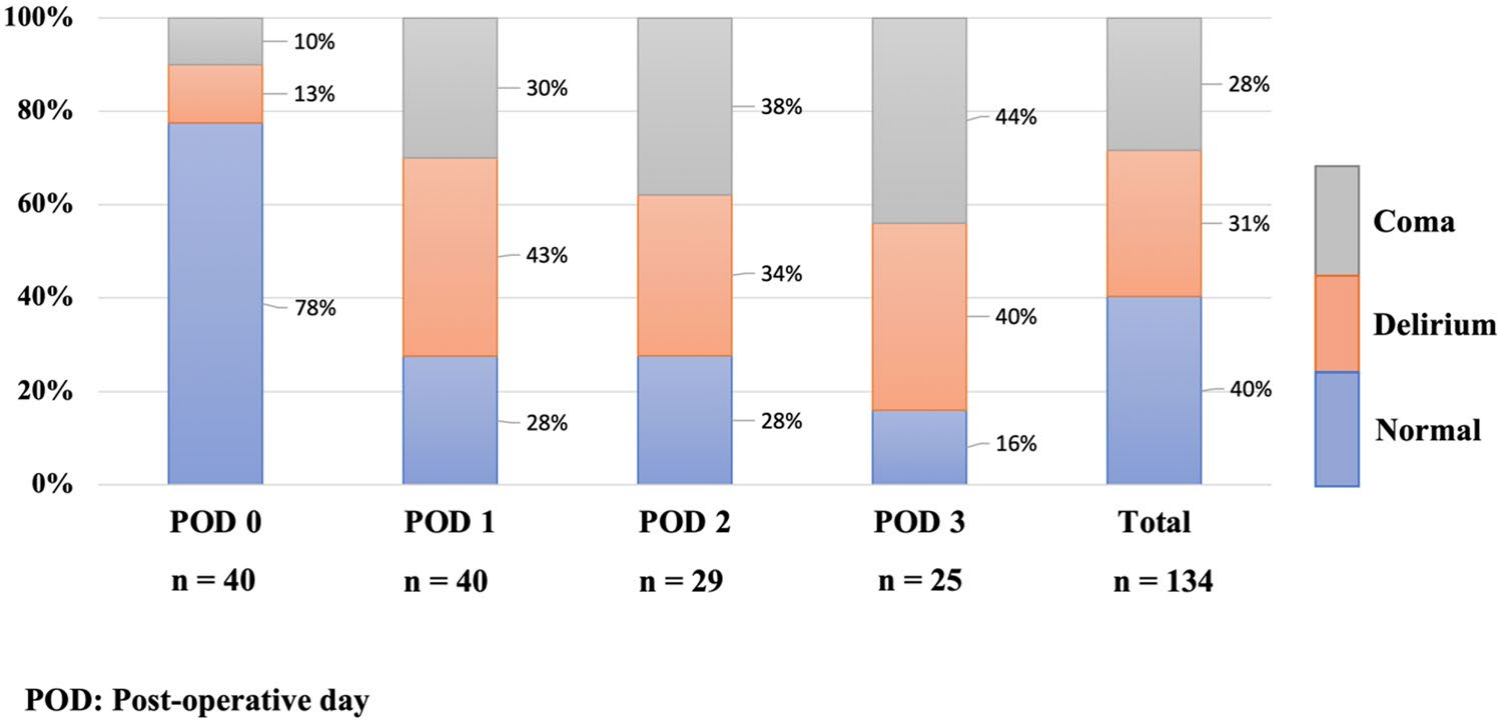
The distribution of post-operative normal, coma, and delirium states.

With regard to conscious status, 78% of patients were ranked as comatose at POD:0 and this gradually decreased to 16% but delirium increased at POD:1 (43%) compared to POD:0 (13%) and this proportion of delirium was maintained until POD:3 (40%) (Fig. 1).

**Figure 2:**
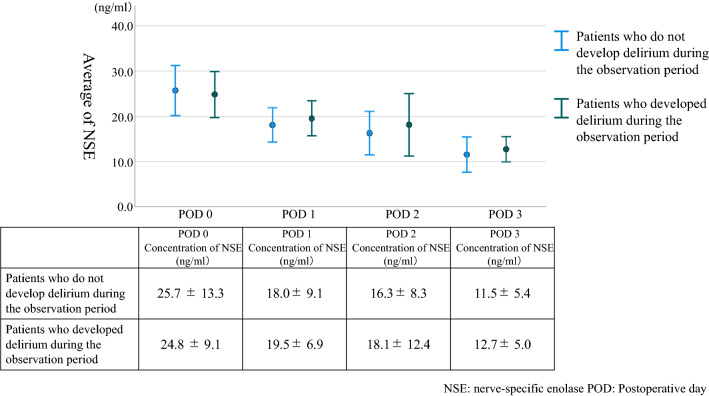
Daily concentrations of NSE. The figure shows the transition of daily concentrations of NSE between groups that experienced delirium during the observation period and those that did not.

Concentrations of NSE gradually decreased after surgery (Fig. 2). In patients who did not develop delirium during the observation period, the average blood concentration of NSE was 25.7 (± 13.3) on POD0, decreasing to 11.5 (± 5.4) by POD3. However, in patients who developed delirium during the observation period, the average blood concentration of NSE was 24.8 (± 9.1) on POD 0 but this decreased to 12.7 (± 5.0) by POD3 (Fig. 2).

### Multivariate modeling

Rhat for all parameters was less than 1.1 and MCSE/sd was less than 10%. Therefore, our model did not violate any assumptions and indicated a good fit for estimation. We show the result of our multi-regression model in Table 2. In this model, NSE was positively associated with coma [posterior odds ratio (OR) = 1.1, 95% credible interval (CrI) 1.01–1.19] while pSOFA [posterior OR = 1.63, 95% CrI 1.17–2.5], midazolam [posterior OR = 1.02, 95% CrI 1.01–1.04], and dexmedetomidine [posterior OR = 9.52, 95% CrI 1.02–108.85] were also positively associated with coma. The association of other factors, including age [posterior OR = 1, 95% CrI 1–1], sex [posterior OR = 2.38, 95% CrI 0.5–13.32], RACHS-1 [posterior OR = 0.64, 95% CrI 0.15–2.38], and fentanyl [posterior OR = 1.64, 95% CrI 0.19–11.58] were of uncertain effect in our model. Results from our univariate modeling are shown in Table 2.

**Table 2:** Regression model for hierarchical consciousness status.

Variable	Multivariate Posterior OR (95% Crl)	
Age	1	(1–1)
Sex	2.38	(0.5–13.32)
RACHS-1	0.64	(0.15–2.38)
NSE	1.1	(1.01–1.19)
pSOFA^a^	1.63	(1.17–2.5)
Dexmedetomidine^b^	9.52	(1.02–108.85)
Midazolam^b^	1.02	(1.01–1.04)
Fentanyl^b^	1.64	(0.19–11.58)

a: Exclude GCS b:μg/day/kg.

*Crl* credible interval, *OR* odds ratio, *RACHS-1* Risk Adjustment in Congenital Heart Surgery, *pSOFA* pediatric Sequential Organ Failure Assessment.

### Confirmation of hierarchical association of conscious status

**Figure 3:**
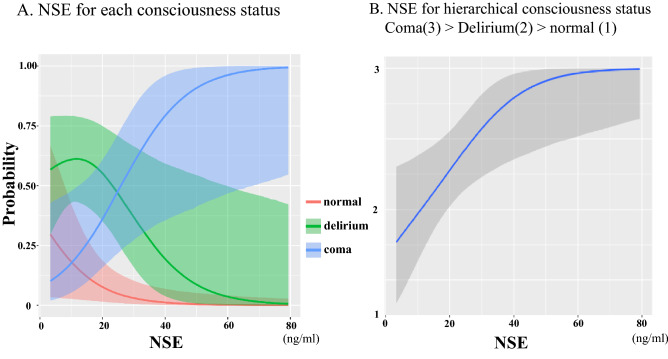
Associations between NSE and conscious status. The figure shows the association between NSE and conscious status in lower concentrations of NSE: normal status is (red line) the highest probability while, at the middle range of NSE, the provability of delirium status (green line) gradually increases. In the situation of higher NSE concentrations, coma status (blue line) has the highest probability. This result indicates that coma, delirium and normal statuses are hierarchically associated with NSE.

We show the estimated associations between NSE and consciousness status in Fig. 3. At lower concentrations of NSE, normal status is the highest probability while the middle range of NSE shows a gradual increase towards delirium status (Fig. 3A). Situations where NSE concentrations were higher saw the highest probabilities of comatose status (Fig. 3A). This result indicated that coma, delirium and normal statuses are hierarchically associated with NSE and coma/delirium are positively associated. The link between NSE and coma according to this hierarchical relationship is shown in Fig. 3B. The association between a hierarchically conscious status and NSE is positive as described above in *Multivariate modeling* [posterior OR = 1.1, 95% CrI 1.01–1.19]*.*

### Associations between the CAPD scores and NSE

Associations between the CAPD scores and NSE are shown in Table 3 which demonstrates the links without coma data. NSE is positively associated with CAPD scores [posterior odds ratio OR = 1.32, 95% CrI 1.09–1.58].

**Table 3:** Regression model for the CAPD scores.

Variable	Multivariate Posterior OR (95% Crl)	
Age	1	(1–1)
Sex	1.08	(0.01–90.01)
RACHS-1	1.16	(0.02–56.26)
NSE	1.32	(1.09–1.58)
pSOFA^a^	4.26	(1.61–11.58)
Dexmedetomidine^b^	192.58	(0.15–90,219)
Midazolam^b^	1.05	(1.01–1.09)
Fentanyl^b^	8.33	(0.12–651.97)

a: Exclude GCS b:μg/day/kg.

*CAPD* Cornell Assessment of Pediatric Delirium, *Crl* credible interval, *OR* odds ratio, *RACHS-1* Risk Adjustment in Congenital Heart Surgery, *pSOFA* pediatric Sequential Organ Failure Assessment.

### Correlation between NSE and pain, delirium, and withdrawal symptoms

Finally, we conducted a correlation analysis between NSE and pain, delirium, and withdrawal symptoms. Of all the components of each symptom, lack of eye contact was the most correlated with NSE (lack of eye contact: r = 0.55). However, all components were less-than-strongly associated (less than 0.7). The full result of this correlation analysis is shown in Table 4.

**Table 4:** Correlation between NSE and pain, delirium, and withdrawal symptoms.

Variable	Correlation analysis	*p*-value
**Pain: FLACC behavior scale**		
Face	0.15	0.17
Legs	0.17	0.11
Activity	0.27	0.01
Consolability	0.06	0.58
Cry	0.31	< 0.01
**Iatrogenic withdrawal syndrome: WAT-1**		
Any loose /watery stools	0.08	0.46
Any vomiting/wretching/gagging	0.01	0.86
Temperature > 37.8 °C	0.16	0.13
State	0.33	< 0.01
Tremor	0.09	0.38
Any sweating	0.04	0.71
Uncoordinated/repetitive movement	0.25	0.02
Yawning or sneezing	0.1	0.34
Startle to touch	0.16	0.14
Muscle tone	0.15	0.18
Time to gain calm state	0.24	0.03
**Delirium: CAPD**		
Eye contact	0.55	< 0.01
Action	0.42	< 0.01
Aware	0.49	< 0.01
Communicate	0.25	0.02
Restless	− 0.07	0.51
Inconsolable	− 0.003	0.98
Underactive	0.25	0.02
Respond	0.2	0.07

*CAPD* Cornell Assessment of Pediatric Delirium, *FLACC* Face, Leg, Activity, Consolability behavioral scale, *WAT-1* Withdrawal assessment tool.

## Discussion

This prospective study aimed to evaluate associations between PD and the representative brain injury biomarker NSE with 134 serum samples and assessment scoring from 40 pediatric patients in the PICU after cardiac surgery. The present study is the first to demonstrate that PD was associated with increased NSE after cardiac surgery and confirmed the concept of hierarchical consciousness status (coma, delirium and normal) in Japanese pediatric patients based on NSE.

Several previous studies showed frequent incident delirium after cardiac operations in adult^1^ and pediatric patients^48^ as these reports argued that delirium is a form of brain dysfunction. Additionally, cardiac surgery is highly invasive, and a previous study showed that brain injury biomarkers, such as NSE, are a consequence of using cardiopulmonary bypass^18^. This putative association asserts that brain injuries are caused by cardiac surgery and delirium is likely to emerge in post-surgical recovery because of depressed brain function. Our present results with hierarchical Bayesian modeling support this concept.

**S**everal studies recognized delirium as a biomarker of acute brain dysfunction in adults^49^. Reflecting this phenomenon, delirium affects cognitive dysfunction after 3 months^50^ and meta-analysis also shows a rigorous association between delirium and long-term cognitive decline^51^. However, while there are still limited reports of long-term outcomes in pediatric patients, this study shows a probable association between delirium and the brain biomarker NSE. Indeed, even though S-100β is thought to be a major potential biomarker associated with delirium in adults, we used NSE for this study as a previous fundamental study clearly showed longer-duration time course changes in S-100β after hypoxia.

We assumed the consciousness status as hierarchical (coma, delirium and normal as ordered) based on the current literature of delirium as a form of brain dysfunction representative of the concept of delirium coma free days (DCFDs)^5,6^. However, confirmation of this association has not been performed even in adult patients and, thus, no standard methodology exists for verification. However, under the hypothesis of the hierarchical stratification of consciousness, models, such as the proportional odds regression model, require that the odds ratio must consistently increase or decrease (parallel line test assumption). In a similar manner, we were able to employ the parallel line test assumption in our model by using hierarchical Bayesian modeling to simulate the probability of each NSE concentration value and verified that consciousness can be hierarchically sorted into normal, delirium and coma statuses. From the perspective of NSE as a brain injury biomarker, our data indicate that delirium is a transitional state of physiological, injury-induced brain dysfunction that exists between normal and comatose statuses. Our model supports this assertion by showing that CAPD scores correlate with serum NSE levels and such a robust association with CAPD results aptly describes brain dysfunction.

We also conducted a correlation analysis of each component of pain, withdrawal and delirium, and found that “eye contact” and “aware of surroundings” were the most correlated with NSE concentration. Similarly, a previous study on children in the early postoperative period evaluated the distinctiveness of delirium and pain after operation and “No eye contact” and “no awareness of surroundings” were highly associated with positive delirium assessments while “Abnormal facial expression”, “crying”, and “inconsolability” were highly associated with positive pain assessments^52^. However, as that study only compared positive delirium assessments on a component level, its still-important findings only revealed the internal validity of the PD and pain scales. Our study thus extends the previous findings by setting brain injury as the external quotative indicator and defining two components from delirium as highly correlated. “Eye contact” is also a key component of two other delirium assessment scales: The Preschool Confusion Assessment Method for the ICU (psCAM-ICU)^8,53,54^ and Sophia Observation withdrawal Symptoms-Paediatric Delirium scale (SOS-PD)^55^.

We used CAPD for PD after cardiac surgery as a single center study, verifying our method successfully within this prospective analysis. Therefore, further studies should be conducted as multicenter or expanded types to reveal the associations between brain injury markers and delirium in general pediatric surgery.

To conclude, the brain injury marker NSE and pediatric delirium are associated, with eye contact as the most predictive factor for increased brain injury as indicated by several delirium assessment tools. Post-intensive care syndrome, especially with regard to cognitive dysfunction, has been recently acknowledged as a problem for pediatric intensive care patients^56^. We believe our findings contribute to the establishment of a brain-protective strategy for post-cardiac pediatric patients through careful bedside monitoring.

## Limitation

Limitations to this study exist. This report was based on only a single-center study, but we believe our patient pool was of sufficient number and power to maintain validity for our main result. Next, as all patients were below grade 3 by RACHIS-1 standards (excluding patients that received more severe operations), this may reduce the generality of our findings. However, most severe operation cases use muscle relaxants, which may confound results, to avoid severe heart failure and reduce oxygen consumption. Moreover, most of the patients in this cohort used benzodiazepines as a primary sedative and this may have caused bias in the relationship between NSE and PD. In spite of these limitations, this study still demonstrates an association between brain injury biomarker NSE and PD that could serve to bring insight into post-cardiac surgery management.

## Conclusion

PD within a hierarchical consciousness status and CAPD scores are associated with brain injury marker NSE. Moreover, eye contact moderately correlates with increased brain injury as several delirium assessment tools emphasize. We would thus like to encourage use of delirium assessment tools to monitor for brain injury, especially in patients who cannot otherwise communicate.

## Data Availability

The datasets used and/or analyzed during the present study are available from the corresponding author upon reasonable request.
